# The Effect of Ketogenic Diet on Inflammatory Arthritis and Cardiovascular Health in Rheumatic Conditions: A Mini Review

**DOI:** 10.3389/fmed.2021.792846

**Published:** 2021-12-14

**Authors:** Jacopo Ciaffi, Dmitri Mitselman, Luana Mancarella, Veronica Brusi, Lucia Lisi, Piero Ruscitti, Paola Cipriani, Riccardo Meliconi, Roberto Giacomelli, Claudio Borghi, Francesco Ursini

**Affiliations:** ^1^Medicine and Rheumatology Unit, Istituto di Ricovero e Cura a Carattere Scientifico (IRCCS), Istituto Ortopedico Rizzoli, Bologna, Italy; ^2^Department of Medical and Surgical Sciences, Istituto di Ricovero e Cura a Carattere Scientifico (IRCCS) S.Orsola, University of Bologna, Bologna, Italy; ^3^Rheumatology Unit, Department of Biotechnological and Applied Clinical Sciences, University of L'Aquila, L'Aquila, Italy; ^4^Department of Biomedical and Neuromotor Sciences (DIBINEM), Alma Mater Studiorum University of Bologna, Bologna, Italy; ^5^Unit of Allergology, Immunology, Rheumatology, Department of Medicine, Università Campus Bio-Medico Di Roma, Rome, Italy

**Keywords:** ketogenic, diet, inflammatory, arthritis, rheumatoid, psoriatic, ankylosing spondylitis, cardiovascular

## Abstract

The principle of ketogenic diet (KD) is restriction of carbohydrates to a maximum of 5–10% of the total daily caloric intake, aiming at shifting body metabolism toward ketone bodies. Different studies suggested promising results of KD to help patients to lose weight, to reduce insulin requirements in diabetes, to supplement cancer protocols, to treat neurological conditions and to optimize control of metabolic and cardiovascular diseases. However, literature about the anti-inflammatory properties of KD in rheumatic diseases is still limited. The beneficial effects of weight loss in patients with inflammatory arthritis can be explained by biomechanical and biochemical factors. Obesity is associated with macrophage activation and production of pro-inflammatory cytokines including TNF-α, IL-1b, and IL-6. The clinical effect of KD may be primarily attributed to improvement of insulin sensitivity. Insulin resistance is associated with an increase of TNF-α, IL-1α, IL-1β, IL-6, and leptin. Moreover, reduction of body's adipose tissue and weight loss account for part of the anti-inflammatory effects and for the impact of KD on cardiovascular health. In rheumatoid arthritis, fasting was shown to be effective in reducing disease symptoms, possibly through the production of β-hydroxybutyrate (BHB), the main ketone body. BHB may exert inhibitory effects also on IL-17 and intermittent fasting improved the clinical manifestations of psoriatic arthritis. In ankylosing spondylitis, current literature doesn't allow to draw conclusion about the effects of KD. Future prospective studies will be needed to elucidate the potential beneficial effects of KD on specific domains and clinical outcomes in patients with inflammatory arthritis.

## Introduction

Ketogenic diet (KD) is characterized by marked carbohydrate restriction, usually to <50 grams a day, and not in a single meal. In a standard KD, carbohydrates should represent about 5–10% of the total daily caloric intake, while the rest of macronutrients consists of proteins (20%) and fats (70–75%) (1). The concept of KD was proposed for the first time in 1921 as a substitute for fasting (2). In the early 20th century, before the introduction of anti-epileptic drugs, fasting was the method of choice to manage epilepsy (3). But fasting, although efficient, cannot be maintained for a long period of time. Therefore, in 1921, Dr. Wilder proposed KD as a suitable method to induce a metabolic state similar to fasting, through the production of ketone bodies, but without caloric restriction. This method was widely used as a treatment for epilepsy during the fourth and fifth decade of 20th century but then dramatically decreased when new anti-epileptic drugs were introduced. KD experienced a reemergence in recent years as a means for weight loss and the physiological concepts behind the dietary regimen gained new scientific interest (3). In the present mini review, we summarize available literature regarding the potential role, pathophysiology and clinical implications of KD in inflammatory arthritis.

Literature review was limited to published primary research, including basic science, cohort studies, intervention and observational trials, and review articles indexed in PubMed.

The following search terms were used: “ketogenic diet” AND “arthritis” OR “rheumatoid arthritis” OR “psoriatic arthritis” OR “ankylosing spondylitis.” As the intent of the review was narrative, inclusion was based on relevance, as deemed so by the authors, to one of the three subcategories of interest: (1) ketogenic diet in rheumatoid arthritis (RA); (2) ketogenic diet in psoriatic arthritis (PsA); (3) ketogenic diet in ankylosing spondylitis (AS). Additionally, articles reporting the effects of KD on cardiovascular health in patients with rheumatic diseases were considered relevant.

### Physiological Effects of Ketogenic Diet

The balance between formation (ketogenesis) and degradation (ketolysis) controls circulating levels of ketone bodies, in a process mainly regulated by the secretion of insulin and glucagon (4). Among the important physiological changes induced by KD there is insulin reduction (5) and the hormonal changes caused by KD, with decreased insulin and increased glucagon levels, favor gluconeogenesis. Under conditions of marked carbohydrate restriction, the body primary energy source switches from glucose toward ketones and fatty acids which are obtained from dietary fat and proteins but also from endogenous sources such as glycogen and adipose stores through lipolysis (6). The accelerated mobilization rate of fatty acids from adipose tissue leads to conversion of acetyl-CoA into ketone bodies, in a process known as ketogenesis (7). Ketogenesis takes place primarily in the liver, which can produce ketone bodies in two ways (8). The first way is the oxidation of fatty acids to acetyl-CoA, which are the building blocks of the ketone bodies, or by conversion of amino acids directly into ketone bodies (9). This results in the synthesis of β-hydroxybutyrate (BHB), acetoacetate and acetone. BHB is the primary and most abundant ketone body found in bloodstream. However, the liver cannot use ketone bodies due to the lack of the enzyme succinyl CoA:3-ketoacid CoA transferase. Therefore, ketone bodies are utilized as a fuel by extra-hepatic tissues, thus sparing glucose metabolism. In these circumstances, ketone bodies replace most of the glucose required by the brain, while liver gluconeogenesis provides the limited amount of energy needed by glucose-dependent tissues such as red blood cells, retina and renal medulla (7).

The mean time for achieving nutritional ketosis is generally 3 days, although longer periods up to 10 days have been documented (10). During the first 3 days, the ketosis induction phase, the patient may experience adverse effects including headache, nausea, asthenia, fatigue, constipation. These effects tend to disappear at the end of the induction phase (10). A different type of adverse effects may be seen in long-duration KD, including gastroesophageal reflux disease, uric acid increase, electrolyte imbalance and hyperlipidemia (11).

### Clinical Effects of the Ketogenic Diet

Insulin reduction and improvement of insulin sensitivity contribute to the clinical effects of KD. Insulin resistance is an impaired response to insulin stimulation of target tissues, primarily liver, muscle and adipose tissue (12). Insulin signal is conducted through complex intracellular mechanisms regulated by multiple kinase enzymes (13). One of the most prominent applications of KD is for the purpose of weight loss (14, 15) and a meta-analysis of 60 dietary trials of weight loss suggested insulin reduction as a *primum movens* of weight loss (16). Another clinical manifestation of KD related to glucose metabolism and insulin regulation can be observed in patients diagnosed with glucose transporter 1 (GLUT1) mutation, which have impaired absorption of glucose from the blood, leading to a reduced supply of nutrients to the brain that manifests as epilepsy. In these patients, KD caused a significant improvement of seizure frequency (17). Furthermore, KD has been evaluated as part of cancer treatment protocols in brain, colon, breast, and lung tumors. These studies resulted in beneficial effects when used alongside chemotherapy, radiotherapy, or both (9). Additionally, KD can be used in the setting of Alzheimer's disease, with an increase in cognitive function (18), and therapeutic effects have been proposed also for neurologic conditions characterized by substantial motor dysfunction (19).

### Ketogenic Diet and Systemic Inflammation

**Figure 1:**
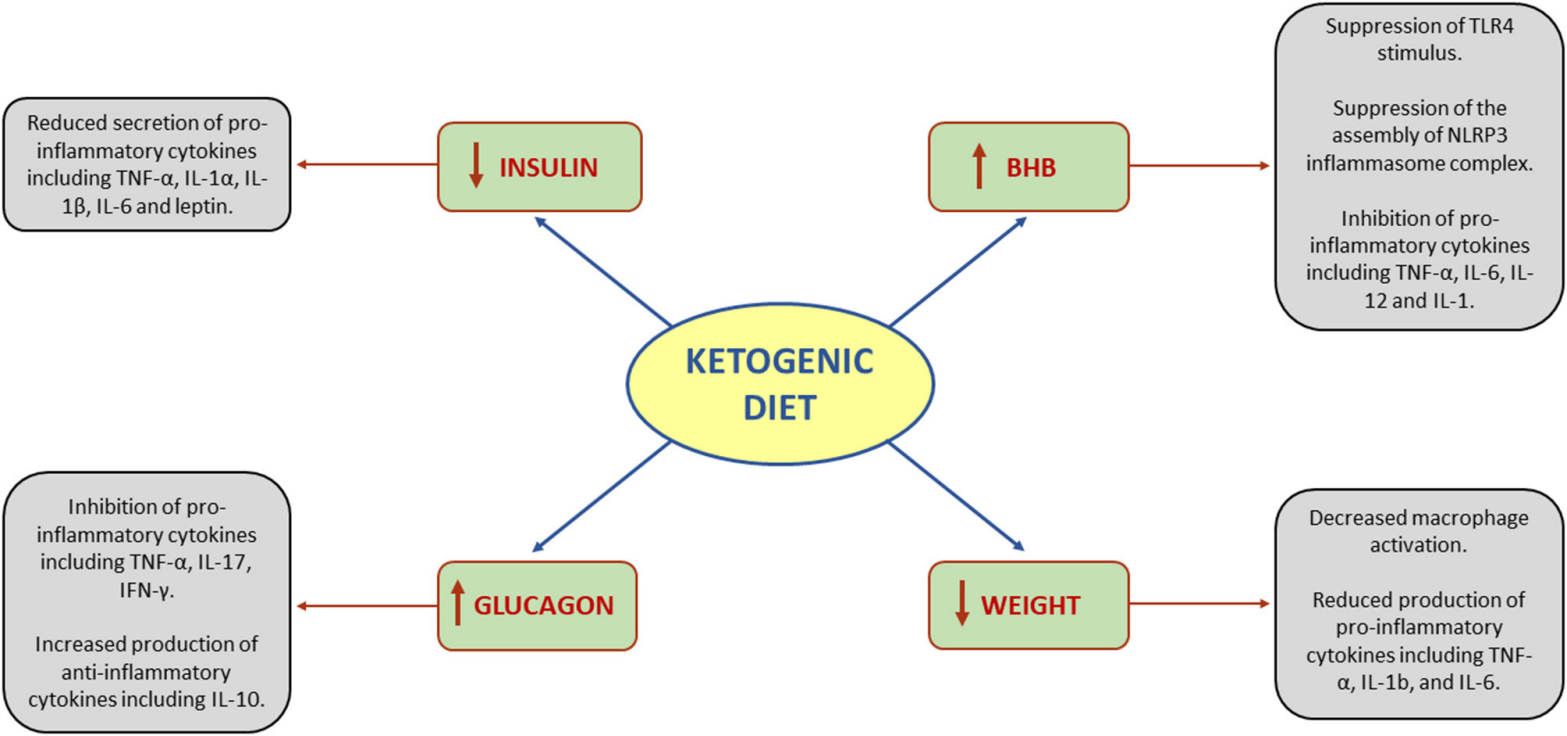
Effects of ketogenic diet on systemic inflammation. BHB, beta-hydroxybutyrate; IFN-γ, interferon gamma; IL, interleukin; NLRP3, NOD-, LRR-, and pyrin domain-containing protein 3; TLR4, toll-like receptor 4; TNF-α, tumor necrosis factor-alpha.

Systemic inflammation is regulated by the production of pro- and anti-inflammatory cytokines. Alterations in the balance of these mediators results in reduction or increase in systemic inflammation (20). The effects of KD on systemic inflammation are related to three main drivers: (1) insulin reduction, (2) BHB synthesis, (3) glucagon increase (21). Additionally, since insulin reduction leads to weight loss, all the anti-inflammatory effects of weight loss should be taken into account as well (Figure 1).

#### Insulin

Insulin and chronic hyperinsulinemia are associated with an increase of the pro-inflammatory cytokines TNF-α, IL-1α, IL-1β, IL-6, and leptin (22). Leptin is a unique cytokine due to its adipose-derived origin. Adipose tissue is considered not only as the body's energy reservoir, but also as an endocrine tissue that can produce proinflammatory cytokines (23). Furthermore, weight loss is a relevant part of the anti-inflammatory effects of KD and reduction of body's adipose tissue has an influence on the secretion of hormones such as leptin (24).

#### β-Hydroxybutyrate

BHB has a double effect on NLRP3 inflammasome complex (NIC). NIC is a protein complex involved in monocyte-induced inflammation (25). When activated, NIC functions as an inducer of caspase 1, which cleaves pro-IL-1β. After being cleaved, pro-IL1β becomes functional IL-1β (26). The activation of NIC requires two steps: first the stimulus from Toll-like receptor 4 (TLR4) that promotes the synthesis of NIC proteins, and then a second step which is their assembly. BHB suppresses TLR4 stimulus and the assembly of NIC individual proteins (27). Furthermore, BHB functions as a ligand to hydroxycarboxylic acid receptors (HCAr). HCAr, also known as GPR109a, is a G protein-coupled receptor predominantly expressed in adipose and immune cells (28). Activation of HCAr suppresses pro-inflammatory cytokine production, including TNF-α, IL-6, IL-12, and IL-1 (29).

#### Glucagon

The increase in glucagon is a direct effect of the decrease in insulin. Nevertheless, glucagon is also a potent hormone that affects many systems, including the immune system (30). Glucagon exerts its effect by activating the cyclic adenosine monophosphate (cAMP) pathway. The effects of cAMP activation vary between tissues and even between cells of the same tissue. In dendritic cells, the increase in cAMP suppresses the release of pro-inflammatory mediators including TNF-α, IL-17, IFN-γ, and promotes anti-inflammatory cytokine production such as IL-10 (31). T cells have shown to reduce proliferation and production of IL-2 (31) and to increase production of IL-4 and IL-5, both interleukins that promote Th2 differentiation (32). In smooth muscle cells, cAMP inhibits proliferation and suppresses the release of cytokines such as IL-1ß and IL-8 (30).

#### Weight Loss

The beneficial effects of weight reduction can be explained by biomechanical and biochemical issues. Weight loss reduces the load exerted on joints (33). Increased adipose tissue has been associated with local and systemic inflammation (23). Obesity is implicated in macrophage activation and production of pro-inflammatory cytokines including TNF-α, IL-1b, and IL-6 (34). As mentioned previously, insulin reduction precedes weight loss (16). Therefore, insulin reduction can be considered a benefit of weight loss. Part of insulin pro-inflammatory effect is exerted through inhibition of anti-inflammatory cytokines production (35). KD, besides the potential anti-inflammatory properties, is also the best non-surgical treatment for weight reduction. A meta-analysis of 53 studies including 68.128 participants suggested that higher-fat, low-carbohydrate diet, was the best intervention to achieve the weight loss and weight maintenance targets (36). In RA, it is important to distinguish between intentional and unintentional weight loss. Intentional weight loss is beneficial in improving the clinical aspects of RA, while unintentional weight loss can worsen the manifestation of RA (37). A retrospective analysis of electronic medical records of 178 patients diagnosed with RA demonstrated obese and overweight patients who achieved weight reduction exceeding 5 Kg had a three-fold higher probability to experience improvement in RA symptoms in comparison with patients who did not succeed in losing weight (38). Similar results were observed in PsA, where several disease activity parameters improved after weight loss treatment with very low energy diet, in a dose responder manner (39).

## Ketogenic Diet in Rheumatoid Arthritis

Several studies have been conducted to investigate the role of dietary interventions in RA (40–44). Available evidence suggests the potentially beneficial effects of anti-inflammatory diets on disease activity (45). Products such as red meat, salt or high-fat diet may trigger inflammation, while fruit, vegetables and fish may exert an anti-inflammatory action (46, 47). Nevertheless, there is no specific dietary recommendation in RA.

A systematic review of 70 dietary studies revealed that fasting, omega 3 and vitamin D3 significantly reduced RA symptoms (48). Fasting and calorie restriction, in particular, were associated with improvement of RA activity, with stronger effects on subjective symptoms (49).

During Ramadan, Muslims refrain from eating and drinking from dawn to sunset. It results in a month of intermittent fasting. Studying the effects of fasting during Ramadan, Su et al. (50) observed non-significantly higher DAS-28 scores before than during Ramadan and significant improvement in morning stiffness and functional disability. Current literature suggests the benefit of fasting in treatment of RA (49–52) and, as previously mentioned, the metabolic state induced by fasting can be considered similar to KD. On this basis, it is conceivable a role for KD in RA but the available knowledge outlines little efficacy (53, 54). Fraser et al. (54) found that fasting, but not KD, significantly decreased serum IL-6 levels and improved disease activity in RA patients. However, in these studies KD was protracted only for 7 days, in order to reproduce the effects of fasting. It has been demonstrated that longer periods are needed to have a response on pain control (55) and to negatively affect oxidative stress (56). It is possible that the tested period of treatment was too short to obtain significant results in RA.

KD may affect RA in several different ways. First, BHB suppresses macrophages and neutrophils' synthesis of IL-1 by inhibiting NIC and thus reducing TNF-α (24, 57). Secondly, BHB has been shown to suppress proinflammatory interleukins including IL-1, IL-12, and IL-6 by activation of HCAr (29). Furthermore, BHB may inhibit the release of IL-1β and IL-18 mediated by NLRP3, contributing to the anti-inflammatory role of KD (58).

## Ketogenic Diet in Psoriatic Arthritis

PsA is associated with several metabolic abnormalities. Insulin resistance, hypertension, diabetes, and hyperuricemia are common comorbidities defining the spectrum of the systemic psoriatic disease (59). Literature about the effects of nutritional interventions in PsA is limited (60, 61), with no specific dietary indication for PsA patients. In psoriasis, it has been shown that a ketogenic nutritional regimen led to significant improvement in disease activity indices (62). Through the assessment of nuclear magnetic medicine metabolomic profile, it was also possible to demonstrate a marked amelioration in biochemical parameters indicative of metabolites related to psoriasis (62). It has been suggested that, in psoriatic patients, KD may facilitate weight loss and modulate systemic inflammation resulting in a quick response to systemic therapy (63–65).

We could not retrieve studies about KD in PsA but some information can be derived from a study conducted on PsA patients during the Ramadan fasting (66). Adawi et al. demonstrated that intermittent fasting improved the clinical manifestation of PsA, including PsA disease activity scores, enthesitis and dactylitis. Furthermore, the patients' improvement was independent of changes in the patients' weight (66). PsA has been strongly associated with Th17 and IL-17A increase (67). In addition to the aforementioned KD effects, BHB induces the production of IL-10 *via* dendritic HCAr, resulting in an inhibitory effect on Th17 (68, 69).

## Ketogenic Diet in Ankylosing Spondylitis

Similar to RA and PsA, there is little evidence that specific dietary interventions influence the activity of AS. Macfarlane et al. (70) conducted a systematic review of 16 publications, including 10 full-text articles, to investigate which dietary regimen induced the best clinical results in AS. The authors concluded that reduction in starch intake, exclusion of dairy products, consumption of fish and fish oil or probiotic supplementation did not improve AS symptoms.

Patients affected by AS and, in general, by axial spondyloarthritis (axSpA), are characterized by an increased cardiovascular risk (71–73). Mediterranean diet has been shown to exert a protective role on cardiovascular morbidity (74, 75). When the impact of Mediterranean diet was investigated in axSpA patients, both the subjective perception of pain, acute phase reactants and disease activity improved after 6 months of nutritional intervention (76).

One of the key pathogenetic mechanisms of AS is the impairment of immunomodulatory function of regulatory T cells, resulting in enhanced IL-17 and other pro-inflammatory cytokines production, with proliferation of pro-inflammatory T cell subsets (77). This process leads to inflammation in the enthesis and ileum of patients with active disease (78). Th17 differentiation is facilitated by TGF-β and IL-6, while IL-23 is determinant to stabilize and maintain TH17 activation and secretion of pro-inflammatory cytokines (79).

Interestingly, KD alters the gut microbiome, with ketone bodies directly inhibiting the growth of gut bacteria. Data obtained from mice models suggest that, reducing the colonization levels of gut bacteria, KD may mediate the lack of intestinal Th17 induction (80). KD can thus induce changes in host metabolites. The alteration of gut microbiota may have downstream consequences for immune cells, reducing levels of intestinal TH17 cells (80). Moreover, BHB was shown to affect microbial-mediated immunomodulation in addition to its ability to inhibit the NLRP3 inflammasome with consequent anti-inflammatory effects (80).

## Cardiovascular Risk in Rheumatic Diseases and the Effects of Ketogenic Diet

In RA, the risk of cardiovascular diseases has been reported to be higher than in the general population, in particular for stroke, heart failure, myocardial infarction and atrial fibrillation (81–84). Patients with RA also have increased mortality rate independently from the presence of other cardiovascular risk factors (85). Additionally, high prevalence of cardiovascular comorbidities and increased mortality related to cardiovascular diseases has been demonstrated in patients with PsA and AS (71, 86–90). Robust evidence outlines a pivotal role of inflammation and immune system dysregulation in the pathogenesis of atherosclerosis and endothelial damage (91–93). Biologic and non-biologic anti-rheumatic therapies may exert a protective role on cardiovascular outcomes in patients with rheumatic diseases (94) and optimizing the control of disease activity has been associated with reduction of cardiovascular events in RA, PsA and AS (95–98). Assessment and management of cardiovascular risk factors is therefore essential in the follow-up of patients with rheumatic diseases. Although no study analyzed the effects of KD on cardiovascular health of individuals with inflammatory arthritis, the potential applications of KD in modulating cardiovascular risk factors and outcomes have been extensively investigated in non-rheumatic patients. A systematic review and meta-analysis of clinical trials carried out to study the efficacy of low-carbohydrate diet on major cardiovascular risk factors demonstrated significant reduction in body weight, BMI, abdominal circumference, blood pressure, plasma triglycerides, fasting plasma glucose, glycated hemoglobin, plasma insulin and plasma C-reactive protein, along with an increase in HDL-cholesterol (99). An overall beneficial impact of low-carbohydrate diet on cardiovascular health was therefore observed, although a possible duration effect was suggested, with benefits that decrease over time. However, other reviews found controversial results, with no or little difference in changes of cardiovascular risk factors with KD, rising also questions about prolonged adherence to the dietary regimen (14, 100). In conclusion, current literature suggests that KD might be associated with improvement of cardiovascular risk factors, mainly driven by weight loss possibility but further studies are needed to evaluate the long-term effects of KD on cardiovascular outcomes and also to assess which is the optimal macronutrients composition (101).

## Discussion

KD is a well-established treatment option used since the 1920s for drug-resistant epilepsy (102). Emerging evidence suggests an adjuvant role of KD in cancer treatment (9, 103) and a possible applicability also in other conditions such as Alzheimer's disease (18) and Parkinson's disease (104). However, the most significant results of KD have been obtained in treating obesity, with robust evidence showing improvement in body weight and reduction in levels of cholesterol, triglycerides and blood glucose (4, 14, 105). Moreover, a role of KD in the reduction of cardiovascular risk has been proposed (99).

Obesity has been proposed as an environmental factor promoting onset and evolution of autoimmune diseases through a direct involvement of adipokines in their pathogenesis (106). Common mechanistic pathways are shared by obesity and rheumatic conditions (107) and the assessment of obesity status and of possible therapeutic interventions is of relevance when establishing a treatment plan for patients with rheumatic and musculoskeletal diseases (108). Extensive experimental and clinical research suggests that being overweight or obese impacts not only disease activity but also several aspects of the life of patients living with inflammatory arthritis (109). Weight loss improves outcomes in patients with RA (37, 38, 44, 49), PsA (39, 61, 64) and AS (110, 111). Besides inflammatory arthritis, metabolic, and eating disorders were suggested to facilitate the occurrence of early clues of connective tissue disorders (112) and obesity was shown to worsen musculoskeletal symptoms also in patients with fibromyalgia (113). Promoting weight loss strategies may be part of the treatment for overweight and obese patients with fibromyalgia but evidence regarding the efficacy of KD in this setting is lacking. Obesity is also a known risk factor for development and progression of osteoarthritis (114). Weight-loss programs were shown to ameliorate osteoarthritis symptoms (115), with exercise and diet therapy leading to significant improvements (116). Low-carbohydrate diet reduced pain intensity in individuals with knee osteoarthritis (117) but, again, the efficacy of a therapeutic KD on different domains of osteoarthritis such as symptoms, pain relief, physical function and health-related quality of life has not been evaluated.

In conclusion, literature about the effects of KD on disease activity and patient reported outcomes in inflammatory arthritis is extremely limited. Evidence derived from fasting studies suggests a mild beneficial effect. Since fasting and KD induce a similar metabolic state, a potential efficacy of KD could be assumed but the available data do not allow to draw conclusions. Future prospective, population-based and adequately powered studies of dietary intervention are required to determine whether KD plays a role in the treatment strategy of patients with rheumatic musculoskeletal diseases.

## Author Contributions

All authors listed have made a substantial, direct, and intellectual contribution to the work and approved it for publication.

## Funding

This work was supported by progetto 5x1000 anno 2019 (redditi 2018) and project 5x1000 year 2019 (income 2018).

## Conflict of Interest

The authors declare that the research was conducted in the absence of any commercial or financial relationships that could be construed as a potential conflict of interest. The handling editor declared a shared affiliation with several of the authors JC, LM, VB, LL, RM, and FU at time of review.

## Publisher's Note

All claims expressed in this article are solely those of the authors and do not necessarily represent those of their affiliated organizations, or those of the publisher, the editors and the reviewers. Any product that may be evaluated in this article, or claim that may be made by its manufacturer, is not guaranteed or endorsed by the publisher.
